# Molecular detection of *P*. *vivax* and *P*. *ovale* foci of infection in asymptomatic and symptomatic children in Northern Namibia

**DOI:** 10.1371/journal.pntd.0007290

**Published:** 2019-05-01

**Authors:** Daniel H. Haiyambo, Petrina Uusiku, Davies Mumbengegwi, Jeff M. Pernica, Ronnie Bock, Benoit Malleret, Laurent Rénia, Beatrice Greco, Isaac K. Quaye

**Affiliations:** 1 Department of Biochemistry and Microbiology, University of Namibia School of Medicine, Windhoek, Namibia; 2 National Vector Borne Disease Control Program, Ministry of Health and Social Services, Windhoek, Namibia; 3 Multidisciplinary Research Center, University of Namibia, Windhoek, Namibia; 4 Division of Infectious Disease, Department of Pediatrics, McMaster University, Hamilton, Ontario, Canada; 5 Department of Biology, University of Namibia, Windhoek, Namibia; 6 Department of Microbiology and Immunology, Yong Loo Lin School of Medicine, National University of Singapore, National University Health System, Singapore; 7 Singapore Immunology Network (SIgN), Agency for Science, Technology and Research (A*STAR), Biopolis, Singapore; 8 Research and Development Access, Global Health Institute, Merck KGaA, Darmstadt, Germany; Eijkman-Oxford Clinical Research Unit, INDONESIA

## Abstract

**Background:**

Knowledge of the foci of *Plasmodium* species infections is critical for a country with an elimination agenda. Namibia is targeting malaria elimination by 2020. To support decision making regarding targeted intervention, we examined for the first time, the foci of *Plasmodium* species infections and regional prevalence in northern Namibia, using nested and quantitative polymerase chain reaction (PCR) methods.

**Methods:**

We used cross-sectional multi-staged sampling to select 952 children below 9 years old from schools and clinics in seven districts in northern Namibia, to assess the presence of *Plasmodium* species.

**Results:**

The median participant age was 6 years (25–75%ile 4–8 y). Participants had a median hemoglobin of 12.0 g/dL (25–75%ile 11.1–12.7 g/dL), although 21% of the cohort was anemic, with anemia being severer in the younger population (p<0.002). Most of children with *Plasmodium* infection were asymptomatic (63.4%), presenting a challenge for elimination. The respective parasite prevalence for *Plasmodium falciparum* (*Pf)*, *Plasmodium vivax (Pv)* and *Plasmodium ovale curtisi* (*Po)* were (4.41%, 0.84% and 0.31%); with Kavango East and West (10.4%, 6.19%) and Ohangwena (4.5%) having the most prevalence. *Pv* was localized in Ohangwena, Omusati and Oshana, while *Po* was found in Kavango. All children with *Pv/Pf* coinfections in Ohangwena, had previously visited Angola, affirming that perennial migrations are risks for importation of *Plasmodium* species. The mean hemoglobin was lower in those with *Plasmodium* infection compared to those without (0.96 g/dL less, 95%CI 0.40–1.52 g/dL less, p = 0.0009) indicating that quasi-endemicity exists in the low transmission setting.

**Conclusions:**

We conclude that *Pv* and *Po* species are present in northern Namibia. Additionally, the higher number of asymptomatic infections present challenges to the efforts at elimination for the country. Careful planning, coordination with neighboring Angola and execution of targeted active intervention, will be required for a successful elimination agenda.

## Author summary

Namibia is a member of the SADC elimination 8 (E8) group with a target to eliminate malaria by 2020. This target stems from years of aggressive interventional strategies that has led to significant reductions in morbidity and mortality. The focus of this strategy is mainly on *Plasmodium falciparum* as the primary parasite species. Foci of transmission is found in the northern border with Angola and Zambia, which also carries the highest population density. Recently as part of the elimination efforts to predict areas likely to have rebound epidemics, three regions Ohangwena, Kavango and Zambezi were identified. In order to affirm these findings and decision-making process for intervention, we assessed the parasite prevalence in 7 northern regional sites for four *Plasmodium* species. We identified *Pv* and *Po curtisi* parasites in Omusati, Ohangwena and Kavango, as well as a significant number of asymptomatic *Pf* and *Pv* infections, part of which may be due to importation from neighboring Angola. As Namibia is targeting elimination by 2020, careful thought and planning will be required to reach the goal.

## Introduction

Progress in malaria elimination efforts have intensified across the Southern African Development Community (SADC) with the aim of elimination by 2030 for most countries [1], [2], [3], [4]. This means a knowledge of current levels of transmission as part of the malariogenic potential is required. The burden of malaria is still a challenge with sporadic epidemic outbreaks within the region annually, due to continual movements within and between countries [2], [5], [6]. So, the major task for these countries is the increased detection of foci of asymptomatic and symptomatic *Plasmodium* species infections with sustained surveillance efforts, for targeted intervention and prevention of epidemic outbreaks [7], [8], [9]. We previously reported on the presence of *P*. *vivax* asymptomatic infections in Botswana which has impacted the malaria elimination agenda for better [10]. Asymptomatic infections are largely submicroscopic under low transmission settings, so they are not seen either by microscopy or rapid diagnostic test (RDT) and form significant reservoirs for reinfections and transmission of the parasite, making elimination a difficult task [11], [12].

Namibia is a member of the elimination eight group (E8) of SADC, which has targeted malaria elimination by 2020 as part of the vision 2030 agenda to mitigate poverty (*WHO*, *World Malaria Report*, *2017*). It has a population of 2.2 million over an area of 0.83 km^2^ [8], [2]. A greater proportion of the population (55%) live in the north, which is traditionally, the main malarious regions in the country [2]. Namibia has achieved significant declines in symptomatic cases of malaria due to effective control methods [8]. However, the continual migration of individuals to and from Angola (a malaria endemic country) in the North, poses a major challenge of importation of parasites, which confounds the efforts at elimination as it creates changes and uncertainties in the disease burden with hidden asymptomatic cases [13], [7]. To assess the risk associated with persistent migrations, continual surveillance to identify the foci and dynamics of asymptomatic and symptomatic *Plasmodium* species infections in the country is critical for targeted interventional approaches [14]. A previous modelling using the Bayesian Model Based Geostatics (MBG) approach predicted that the three districts most likely to experience rebound or epidemics of malaria in the country were: Ohangwena, Kavango and Caprivi that borders Angola and Zambia in the north [1],[15],[16]. Here we report for the first time on the foci of *P*.*vivax* (Pv) and *P*. *ovale curtisi* (Po) asymptomatic and symptomatic infections and current levels of *P*. *falciparum (Pf)* asymptomatic infections in an active survey in the country using PCR (nested and qPCR) a more sensitive molecular method.

## Methods

### Study sites and selection of population

**Fig 1:**
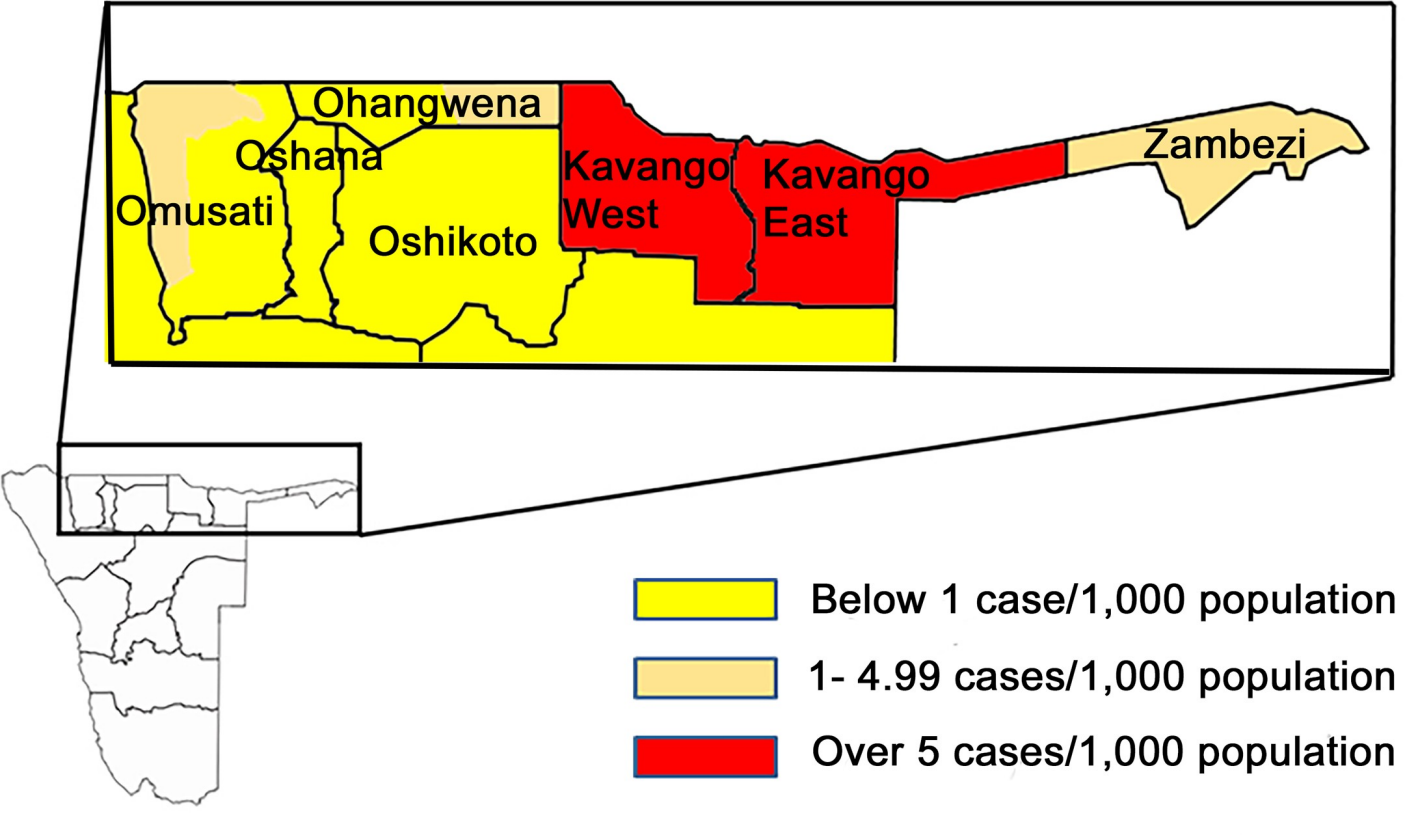
Sampling sites in Northern Namibia. (the color codes coincide with the incidence Map in 2015; *source*: The MAP was obtained from the National Malaria Control Program (NMCP) of Namibia and modified for the manuscript).

The study sites were selected with the support of the National Malaria Control Program (NMCP) of the Ministry of Health and Social Services (MOHSS) and Ministry of Education, Namibia. The sites were: Kunene, Omusati, Oshikoto, Ohangwena, Kavango West, Kavango East and Zambezi (Caprivi) (Fig 1). The sites are color coded to reflect the malaria incidence Map for these sites in 2015, which had not been updated at the time of sample collection. The respective incidence rates were, more than 5 cases per 1000 people in Kavango East and West, 1–4.99 cases per 1000 people in Zambezi, Ohangwena and Omusati, while Oshikoto and Kunene had less than 1 case per 1000 people. Within each district the Ministry of Education primary school registers and MOHSS Clinic/Health post registries were used in a multi-staged sampling process to randomly select the schools and clinics from which the enrolment was done. Details of the sampling procedure are as previously described [10]. Briefly, in the first stage, districts with known malaria transmission profile based on the recommendations of the NMCP were purposely selected. In the second stage, towns within the district with variable malaria incidence rates were also purposely selected from the NMCP recommendations as shown in Fig 1. This was to ensure that areas of high, moderate, low and sporadic transmissions including those closer to the Angolan border were captured in the sampling process. In the third stage, schools and clinics within each town/village were purposely selected using a two-stage clustering approach based on the population density and adequate cross-sectional representation of the communities to avoid any bias. In the final stage, participants were assigned numbers and enrolled based on informed parental consent and consent of heads of schools and clinics. The total number of samples derived for each district/town was in direct proportion to the estimated population density.

### Ethical statement

The study was approved by the MOHSS Ethical Committee, Namibia. All parents/guardians provided informed consent on behalf of all participants. Where needed, assent was also obtained from the child before sample collection.

### Blood sample collection

The study enrolled a total of 952 individuals under 9 years old. Subjects enrolled from schools amounted to 591 while 361 were from Clinics and Health posts. Sampling collection was done from September 2016-October 2017 using a multistage sampling strategy as described previously. Fever was defined as subjects with axillary temperature of ≥ 37.2°C at the time of sample collection, while asymptomatic subjects were those without fever (axillary temperature <37.2°C) and without a history of fever in the preceding 72 hours. Sample collections were timed to cover the onset and peak of the malaria transmission season. Prior to the blood sample being drawn, a short questionnaire was administered for travel history and previous malaria illness in the past year, while basic information on age and sex were documented. An aliquot of 1.5–2.5 ml venous blood was collected into EDTA tubes and centrifuged at 3000 rpm for 5 minutes to separate the buffy coat, plasma and red blood cells into separate tubes. These were then stored at -20°C and later transferred to -80°C till analyzed. Hemoglobin (Hb) was measured using Hemocue (Ängelholm, Sweden).

### Laboratory analyses

#### DNA extraction

Genomic DNA was extracted from pelleted red blood cells using the automated Hamilton Star Microlab Workstation (Hamilton Bonaduz AG, Bonaduz, Switzerland) with the Machery and Nagel 96 blood DNA extraction kit. The principle is based on silica-membrane technology for binding DNA, following lysis to facilitate purification. The starting blood sample was 200 μl of the packed and thawed rbcs, and final DNA elution volume of 120 μl sterile PCR-grade water. 5 μl of the extracted DNA was used for nested PCR while 2.5 μl was used for qPCR.

#### Molecular detection of *Plasmodium* species

All Plasmodium species were detected by a modification of the double nested PCR procedure targeting conserved species-specific regions of the small subunit 18S ribosomal RNA (18S ssRNA) as previously reported [10], [17]. For qPCR the genus specific primers and probes were as previously published [18], while the species-specific primers and probes for *Pf*, *Pv*, *Po* and *Pm*, were those published respectively in the following references [19], [20]. Samples were screened with qPCR for positivity and affirmed with nested for species [17]. All nested detection assays were single-plexed and run in a high throughput 96 well plate Applied BioSystem GeneAmp 9700 PCR system (ABI 9700, Singapore) while the CFX96 Real-Time PCR detection system (Bio-Rad Laboratories, Inc, Pretoria, South Africa) was used for all qPCR assays. The PCR kits were the Qiagen PCR core kit (Qiagen Inc, Valencia, CA) for nested PCR, and the KAPA PROBE FAST qPCR Master Mix (2X) (Cape Town, South Africa) or the abTES Malaria 5 qPCR II Kit (AITbiotech Pte Ltd, Singapore. Species specific primers and probes were ordered from Eurogentec, Liege, Belgium. Standard controls of Plasmodium species 3D7 synchronized cultured parasites, were obtained by cell sorting flow cytometry as described previously [21], (10,000 ring in 50 μl of packed RBCs). All nested PCR amplification reactions were carried out in a total volume of 25 μl and in the presence of 10 mM Tris-HCl, pH 8.3, 4 mM MgCl_2_, 50 mM KCl, 250nM of each oligonucleotide primer, 200 μM of each of the four dNTPs and 0.5 units of Dream Taq DNA polymerase. 2.5 μl of DNA prepared from whole blood was used to initiate the genus specific primary amplification PCR reaction and 2 μl of the product was then used in the secondary amplification including the species-specific detection assays. The PCR cycling parameters for the primary amplification were as follows: initial denaturation at 94°C for 10mins, preceded the cyclin conditions which consisted of denaturation at 94°C for 30 s, annealing at 55°C for 1 min and extension at 72°C for 1 min for 35 cycles. After the final annealing step followed by extension at 72°C for four minutes, the reaction was ended. In the second nested PCR, annealing temperature of 58°C was used. For the species-specific amplifications, the only changes were initial denaturation at 94°C for four minutes and annealing at 58°C. To ensure that there was no cross contamination, negative control samples (no DNA template) were randomly included in the run. The consistency of the results was checked by multiple runs of several subsets of the samples which were randomly picked. The amplified products were analyzed on 1.5% agarose gels by electrophoresis, followed by visualization on a UVP Geldoc-it Imager TS 310 (Cambridge, UK) after ethidium bromide staining. For qPCR, we used KAPA PROBE FAST qPCR Master Mix (2X) (Cape Town, South Africa) kits with our primers and probes, and the *ab*TES Malaria 5 qPCR II Kit. For qPCR 5μl of extracted DNA was used as template in a 25μl qPCR reaction containing 12.5μl KAPA PROBE FAST qPCR Master Mix (2X), 300 nM forward and reverse primer and probe for each species and nuclease free water. The PCR parameters were as follows: 95°C for 2 minutes followed by 45 cycles of amplification (95°C for 5 seconds and 60°C for 20 seconds).

The qPCR assay for *Plasmodium* genus detection was validated using 1 μl of serially diluted *Plasmodium* species 3D7 standards as template for qPCR reactions. Each run included a negative control (nuclease free water) and positive controls (*P*. *falciparum* and *P*. *vivax*). The qPCR procedure for the abTES Malaria qPCR II Kit was done according to the manufacturer’s instructions.

### Statistical analysis

Data were entered in an Excel data sheet and STATA v11.2 (StataCorp, College Station, TX, USA) was used for analysis. Descriptive statistics and appropriate measures of central tendency were provided for relevant demographic covariates. To describe differences between study sub-populations (eg. different regions of residence, presence/absence of *Plasmodium* infection), continuous covariates were compared using linear regression or the student t-test and categorical variables were compared using logistic regression, the Chi square test, or Fisher’s exact test. For logistic regression analyses, odds ratios (ORs) were provided. For all point estimates, 95% confidence intervals (CIs) were provided. Anemia was defined as Hb < 11.0 g/dL. Statistical significance for all comparisons was set at p<0.05 and adjustment was not done for multiple comparisons in this exploratory study. P-values smaller than 0.001 were reported as p<0.001.

## Results

### Study population

A total of 952 children from 7 districts (Fig 1.) were assessed for *Plasmodium* species infection. The median participant age was 6 years (25–75%ile 4–8 years). Participants recruited in Kunene (1.60 years younger, 95%ile 0.91–2.29 years younger), Ohangwena (1.24 years younger, 95%CI 0.74–1.74 years younger), and Omusati (1.20 years younger, 95%ile 0.66–1.75 years younger) were all significantly younger than those participants recruited in Kavango East (p<0.001 for all these comparisons). Overall, 52.6% were female. Participants had a median hemoglobin of 12.0 g/dL (25–75%ile 11.1–12.7 g/dL); 21% of the cohort was anemic. There were significant differences in mean participant hemoglobin levels between different regions; those from Kunene (0.72 g/dL less, 95%CI 0.26–1.19 g/dL less, p = 0.002) and Ohangwena (0.65 g/dL less, 95%CI 0.31–0.99 g/dL less, p<0.001) had lower levels than children from Kavango East.

### Plasmodium species

**Fig 2:**
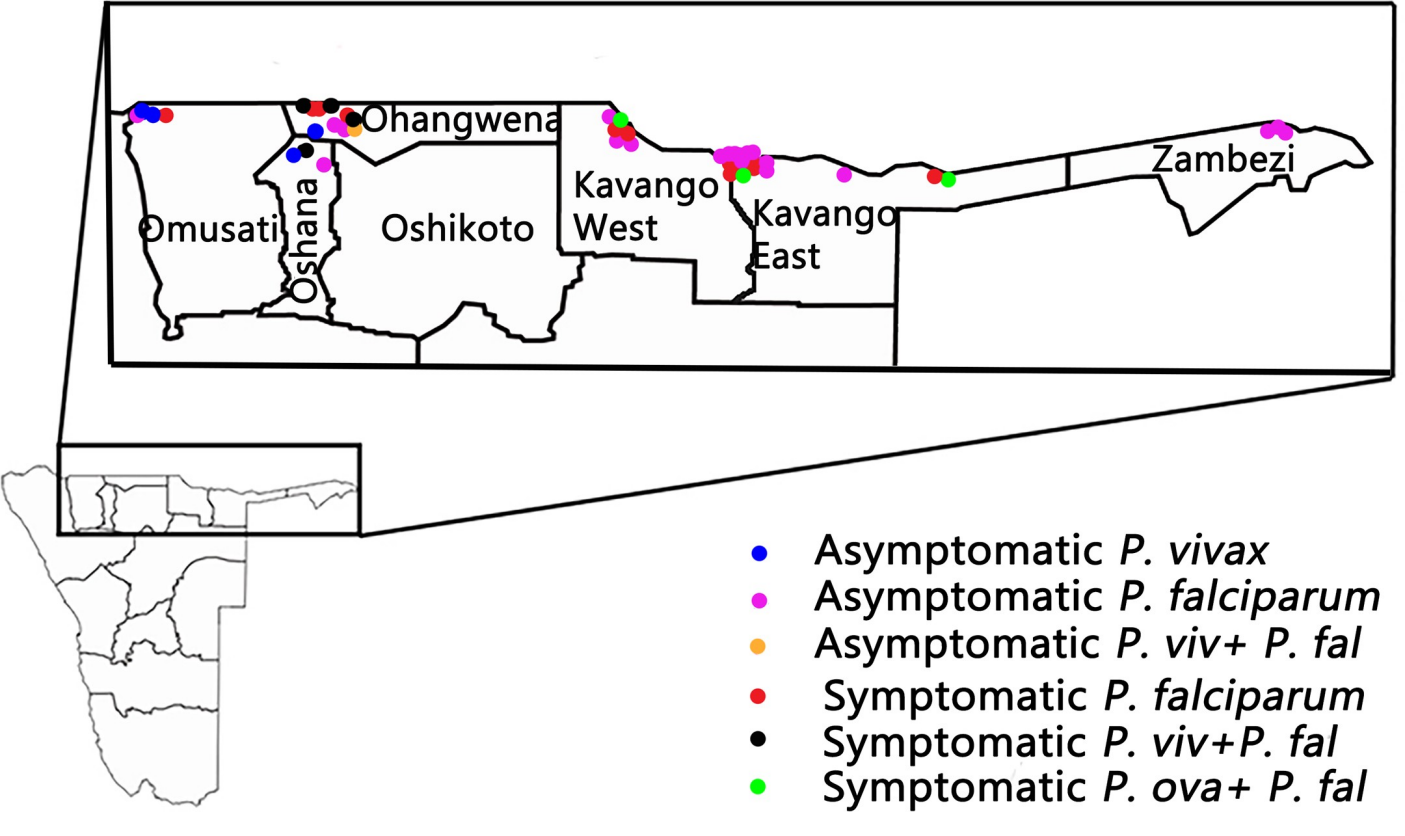
The type and number of plasmodium species detected in asymptomatic and symptomatic subjects. *Note*: This was generated from our data and created using Adobe Photoshop CC 2015.

The majority of *Plasmodium* infections were *Pf* (n = 41), with 8 *Pv* infections, 3 *Po* infections and no *Pm* infections (Fig 2). Coinfections were common; half of the children with *Pv* infections were co-infected with *Pf* and all the children with *Po* infections had *Pf* co-infections. Most of children with *Plasmodium* infection (63.4%) were afebrile. All three children with Pv/Pf coinfections and two with *Pf* infection in Ohangwena, had previously visited Angola. The mean hemoglobin was lower in those with *Plasmodium* infection as compared to those who did not (0.96 g/dL less, 95%CI 0.40–1.52 g/dL less, p = 0.0009). There were no differences in the age or gender distributions for those that did and did not have *Plasmodium* infections. There were clear differences in the proportions of participants infected in the different regions; the highest prevalence was seen in Kavango East (10.4%) and there was a statistically lower prevalence seen in the regions of Kunene (0%, p<0.001), Ohangwena (4.50%, OR 0.40 95%CI 0.17–0.94, p = 0.04), Omusati (2.70%, OR 0.13 95% CI 0.030–0.58, p = 0.007), and Oshana (2.63%, OR 0.17 95%CI 0.039–0.75, p = 0.02). There was no statistical difference between the prevalence of mixed infections in the different regions. Kavango region interestingly, is the most endemic for malaria from the 2015 incidence Map. *Pv* was localized within Omusati, Ohangwena and Oshana regions, while *P ovale* was seen in Kavango. The precise number and type of *Plasmodium* species for each region is presented in Table 1. The overall parasite prevalence was 4.83% with *Pv* and *Po* accounting for 0.84% and 0.31% respectively. The two species therefore account for 1.1% of the parasite prevalence.

**Table 1:** Parasite prevalence of *Plasmodium* species detected per region and overall.

District	Total number of samples tested	Type and number of Plasmodium species identified (overall prevalence)				Total number of Samples from clinics	Total number of Samples from schools	Parasite prevalence/region
		*Pf*	*Pv*	*Pv/Pf*	*Pf/Po*			
Kunene	72	0	0	0	0	45	27	0%
Omusati	148	2	2	0	0	67	81	2.70%
Oshana	114	1	1	1	0	19	95	2.63%
Ohangwena	200	5	1	3	0	90	110	4.50%
Kavango West	97	5	0	0	1	21	76	6.19%
Kavango East	202	19	0	0	2	119	83	10.4%
Zambezi	119	3	0	0	0	0	119	2.52%
**Overall total (rate)**	**952**	**35** **(3.68%)**	**4** **(0.42%)**	**4** **(0.42%)**	**3** **(0.31%)**	**361**	**591**	

## Discussion

The study has demonstrated for the first time that the *Pv* foci of infection in Northern Namibia encompasses three regions: Omusati, Ohangwena and Oshana, while *Po* focus is in Kavango. The study affirms Kavango as remaining a focus for *Pf* infections in northern Namibia, as shown in the incidence map in 2015 and epidemic outbreaks in 2016 [7]. The transmission profile of the parasite shows that *Pv* only infections were all asymptomatic and in a relatively younger population, whereas *Pf* infections were made up of relatively older children and a high number of asymptomatics, spread across all the regions. A similar pattern of *Pv* infection was observed in our previous study in Botswana [10]. Reports from Mali, Senegal, Indonesia, Papua New Guinea (PNG), Brazil and others also indicate that asymptomatic infections are commonly observed [22], [23]. Since the population with *Pv* infections were also anemic compared with those predominantly in Kavango East with *Pf* infection, the findings reveal some basic differences in the infection dynamics of *Pv* and *Pf* in low transmission settings, and in the population in northern Namibia. *Pv* strictly infects reticulocytes [24], so under anemic conditions where increases in reticulocyte counts occur, *Pv* infection will be facilitated. In addition, the relapses associated with the hypnozoites stage of *Pv* infection [25], will increase anti-blood stage immune acquisition more rapidly overtime [26], [27], so that eventually, this will lead to a predominantly asymptomatic population [28], [29]. It is known that all stages of *Pv* infection can develop in the asymptomatic state [30] to sustain transmission. On the other hand, in *Pf* infections where hypnozoite stages are absent, if transmission is persistent, individuals acquire immunity with exposure, so the period of immune acquisition is longer and older people become the carriers of asymptomatic infections [22]. The *Pf* asymptomatic infections point to sustained infections over the years within the population, that has enabled older children to acquire enough immunity to harbor parasites [2]. In a recent MGB modelling method used to predict which regions in Namibia are most likely to experience rebound epidemics, Ohangwena, Kavango and Caprivi (Zambezi) were cited as the major focal areas [7], which is in absolute congruence with the present report. It appears that there is a “quasi-stable” malaria infection scenario within a low transmission setting, where infections although sporadic may be persistent. One should also take cognizance of the fact the there is an inherent genetic variability of parasites for each population that adds to the heterogeneity and so require more tailored interventions, with regards to elimination [31]. The elimination agenda for Namibia has a major challenge of perennial importation of parasite across the border with Angola. This was seen in the present study, with visits to Angola contributing to the *Pv* infections. The challenge significantly complicates the elimination process [1].

There is a need for active systematic investigations and understanding of the epidemiology of asymptomatic malaria of all species in low transmission settings with an elimination agenda. This can initiate in hotspots and hotpops. Asymptomatic malaria sustains malaria transmission all season and so form a formidable component of transmission as they are not targeted for clearance [32]. This could be a major obstacle for elimination in a scenario where the asymptomatic fraction of the population grows rather than diminish with time, as a result of acquired immunity. *Pv* and *Po* hypnozoite forms add to the complexity of dealing with asymptomatic infections with their sequestration in the liver and or bone marrow [33], [22], [34]. So, in low transmission settings, the epidemiology of *Plasmodium* infections can be heterogeneous, requiring a more thorough active assessment in children towards malaria elimination. It is now no longer valid that *Pv* infections do not occur in Africa where Duffy antigen negativity is predominant. Several reports indicate that *Pv* infections occur in Duffy negative individuals in Africa [35], [36]. Clearly, the agenda for malaria elimination should not only be focused on *Pf* infections but done in parallel with non-falciparum malaria.

We conclude that *Pv* asymptomatic infections and *Po* are present in northern Namibia as are asymptomatic infections of *Pf*. These introduce new paradigms in the elimination agenda for Namibia, that requires careful planning and thought for blocking transmission and aggressive targeting of all populations and species affected.

## Supporting information

S1 Checklist STROBE checklist: Attached in the online submission. (DOC)
